# Large-scale deployment of a rice 6 K SNP array for genetics and breeding applications

**DOI:** 10.1186/s12284-017-0181-2

**Published:** 2017-08-30

**Authors:** Michael J. Thomson, Namrata Singh, Maria S. Dwiyanti, Diane R. Wang, Mark H. Wright, Francisco Agosto Perez, Genevieve DeClerck, Joong Hyoun Chin, Geraldine A. Malitic-Layaoen, Venice Margarette Juanillas, Christine J. Dilla-Ermita, Ramil Mauleon, Tobias Kretzschmar, Susan R. McCouch

**Affiliations:** 1 Department of Soil and Crop Sciences, Texas A&M University, College Station, Houston, TX 77843 USA; 2 School of Integrative Plant Sciences, Plant Breeding and Genetics Section, Cornell University, Ithaca, New York, 14853 USA; 3 International Rice Research Institute, Los Baños, Philippines; 4 Present address: Research Faculty of Agriculture, Hokkaido University, Sapporo, Hokkaido 060-8589 Japan; 5 Department of Genetics, Stanford School of Medicine, Stanford, California, 94305 USA; 6 Present address: DeClerck Design, LLC, Freeville, NY USA; 7 Present address: Graduate School of Integrated Bioindustry, Sejong University, 209 Neungdong-ro, Gwangjin-gu, Seoul, 05006 South Korea; 8 Present address: Department of Plant Pathology, Washington State University, Pullman, Washington, 99164 USA

**Keywords:** *Oryza sativa*, *O. rufipogon*, Single nucleotide polymorphism (SNP), High-throughput genotyping, Rice diversity, SNP fingerprinting, CSSL development

## Abstract

**Background:**

Fixed arrays of single nucleotide polymorphism (SNP) markers have advantages over reduced representation sequencing in their ease of data analysis, consistently higher call rates, and rapid turnaround times. A 6 K SNP array represents a cost-benefit “sweet spot” for routine genetics and breeding applications in rice. Selection of informative SNPs across species and subpopulations during chip design is essential to obtain useful polymorphism rates for target germplasm groups. This paper summarizes results from large-scale deployment of an Illumina 6 K SNP array for rice.

**Results:**

Design of the Illumina Infinium 6 K SNP chip for rice, referred to as the Cornell_6K_Array_Infinium_Rice (C6AIR), includes 4429 SNPs from re-sequencing data and 1571 SNP markers from previous BeadXpress 384-SNP sets, selected based on polymorphism rate and allele frequency within and between target germplasm groups. Of the 6000 attempted bead types, 5274 passed Illumina’s production quality control. The C6AIR was widely deployed at the International Rice Research Institute (IRRI) for genetic diversity analysis, QTL mapping, and tracking introgressions and was intensively used at Cornell University for QTL analysis and developing libraries of interspecific chromosome segment substitution lines (CSSLs) between *O. sativa* and diverse accessions of *O. rufipogon* or *O. meridionalis.* Collectively, the array was used to genotype over 40,000 rice samples. A set of 4606 SNP markers was used to provide high quality data for *O. sativa* germplasm, while a slightly expanded set of 4940 SNPs was used for *O. sativa* X *O. rufipogon* populations. Biparental polymorphism rates were generally between 1900 and 2500 well-distributed SNP markers for *indica x japonica* or interspecific populations and between 1300 and 1500 markers for crosses within *indica*, while polymorphism rates were lower for pairwise crosses within U.S. *tropical japonica* germplasm. Recently, a second-generation array containing ~7000 SNP markers, referred to as the C7AIR, was designed by removing poor-performing SNPs from the C6AIR and adding markers selected to increase the utility of the array for elite *tropical japonica* material.

**Conclusions:**

The C6AIR has been successfully used to generate rapid and high-quality genotype data for diverse genetics and breeding applications in rice, and provides the basis for an optimized design in the C7AIR.

**Electronic supplementary material:**

The online version of this article (10.1186/s12284-017-0181-2) contains supplementary material, which is available to authorized users.

## Background

Future challenges to sustainably produce food for 9.5 billion people by 2050 using less land and fewer inputs will require higher yields in intensive systems under increasingly variable environments. Modern plant breeding and genetic research programs aim to utilize the latest technologies to accelerate the annual rate of genetic gain to keep up with rice demand. High-throughput molecular marker techniques have enabled routine, low-cost genotyping for both targeted and genome-wide approaches. Targeted methods, where a few markers (<100) are used to genotype a large number of samples, provide an efficient strategy for forward selection of major genes in breeding programs. Genome-wide methods, including fixed arrays and next generation sequencing, provide marker densities appropriate for genome-wide association studies, QTL mapping, diversity analysis, DNA fingerprinting, impact assessment studies, and breeding applications such as genomic selection (Thomson 2014; Varshney et al. 2014).

Single nucleotide polymorphisms (SNPs) are the markers of choice for most high throughput genotyping applications because they are ubiquitous in eukaryotic genomes, cost-effective to assay using automated platforms, and because allele calling, data analysis and data-basing are straightforward due to their biallelic nature. A number of medium- or high-resolution SNP arrays in rice have been deployed, primarily for genome-wide association studies, including a 44 K SNP chip (Zhao et al. 2011), 50 K SNP chips (Chen et al. 2013b; Singh et al. 2015), and the 700 K high-density rice array (HDRA, McCouch et al. 2016). These arrays provide automated platforms to dissect phenotype-genotype associations, while at the same time offering valuable datasets that can be used to validate high-quality SNP markers that are informative within and between key germplasm groups. The subsequent development of lower resolution detection platforms, including KASP, TaqMan, and Fluidigm that target individual SNPs, and the low-density SNP arrays, have made use of the wealth of information published from the higher-density arrays to extract informative SNPs and invariant SNP flanking sequences that convert well to other assays (McCouch et al. 2010; Tung et al. 2010; Chen et al. 2013a).

Historically, sets of 384, 768, or 1536 SNP markers were used for diversity analysis, QTL mapping, marker-assisted backcrossing, specialized genetic stock development, and pedigree confirmation among breeding lines in rice (Nagasaki et al. 2010; Zhao et al. 2010; Chen et al. 2011; Thomson et al. 2012; Ye et al. 2012; Rahman et al. 2016; Shah et al. 2016). Despite their utility across a variety of applications, the limited numbers of SNP markers in each assay required the development of multiple SNP sets in order to provide a high enough resolution of polymorphic markers for use with specific germplasm groups.

Combining SNP sets into larger arrays helps increase the number of potential users per array, which lowers cost while providing increased resolution across a diversity of germplasm. Previously, an Illumina Infinium 6 K array in rice (RICE6K) was developed in Wuhan, China to provide polymorphic SNPs within and between the *indica* and *japonica* subgroups for applications in background selection, mapping population genotyping, variety identification and purity tests, and bulk segregant analysis (Yu et al. 2013). With rapid genotyping turn-around times, ease of allele calling and data analysis provided by this and other 6 K SNP chips, breeders and geneticists can interact more directly and rapidly with the data and incorporate genotyping results in their programs without dependence upon bioinformatics specialists.

The primary alternative to fixed SNP arrays is reduced representation next-generation sequencing, such as restriction-site associated DNA (RAD) sequencing or genotyping by sequencing (GBS). These methods provide large numbers of genome-wide SNP markers at a low cost (Baird et al. 2008; Elshire et al. 2011; Peterson et al. 2012). While RAD-Seq and GBS have been very successful for certain applications, several limitations have become apparent as adoption has widened. In addition to reliance on complex protocols for library preparation, the requirement to multiplex hundreds of samples to minimize cost, and long delays in obtaining sequencing output, a challenge for many groups has been the costly bioinformatics infrastructure needed to support downstream analytical pipelines for accurate allele-calling. Although imputation techniques enable researchers to fill in gaps in data sets, GBS approaches typically suffer from large amounts of missing data, making it challenging to accurately call heterozygotes. More recently, core facilities are faced with the challenge of dealing with licensing of these technologies due to the KeyGene patent for Sequence-Based Genotyping (Truong et al. 2012; US Patent 8,815,512).

At the other end of the spectrum, targeted simplex SNP approaches, such as TaqMan and KASP-based genotyping, offer an alternative to fixed arrays for applications requiring a few, high-value markers across very large populations (Eathington et al. 2007; He et al. 2014; Semagn et al. 2014). These assays can be cost-effective for large sample sizes (1000 s-10,000 s) and are ideal when trait-predictive SNP markers are available for selection of large effect genes in breeding programs; however, their cost advantage is lost for applications involving small numbers of lines or requiring more than 100–200 genome-wide SNP markers. Thus, while no single genotyping system is ideal for all applications, the wide range of available genotyping platforms now offer solutions that can provide an optimal balance to meet the needs of different users, taking into account cost per sample, marker resolution, turnaround time, allele call rates, and data analysis requirements (Thomson 2014).

To replace multiple rice 384-SNP sets and provide a high-quality set of informative SNP markers for genetics and breeding applications, an Illumina Infinium 6 K SNP chip for rice, referred to as the Cornell_6K_Array_Infinium_Rice (C6AIR), was designed for use at both Cornell University and the International Rice Research Institute (IRRI). The design of the C6AIR includes 1571 SNP markers from legacy BeadXpress SNP sets and 4429 SNPs selected from whole genome re-sequencing data to be polymorphic within and between the target germplasm groups and mapping parents. This paper describes the efficacy of the C6AIR for QTL mapping, genetic diversity analysis, SNP fingerprinting of breeding lines, tracking of introgressions, and checking for recovery of recurrent parent background during marker-assisted backcrossing. Subsequently, an improved second-generation array was developed by removing SNPs that performed poorly on the 6 K array and increasing the number of bead types to just over 7000. This new 7 K rice array, referred to as the C7AIR, provides continuity with data sets from the C6AIR while increasing the number of high quality SNP loci for future use in genetics and breeding applications.

## Results and discussion

### Design of the Cornell 6 K SNP array

The C6AIR was designed and developed to be informative for *Oryza sativa* and *O. sativa*/*O. rufipogon* populations, incorporating markers from previous GoldenGate 384-SNP sets and selective SNPs from whole genome re-sequencing data. The custom-designed Infinium iSelect array consisted of 6000 attempted bead types, including 1571 SNP markers from legacy BeadXpress 384-SNP sets (Thomson et al. 2012) and 4429 SNPs selected from whole genome sequence data to be polymorphic within and between diverse germplasm groups and mapping parents. The re-sequenced genomes used as a SNP discovery dataset were described by McCouch et al. (2016) and included 88 *O. sativa* accessions (21 *indicas*, 16 *aus*, 18 *tropical japonica*, 19 *temperate japonica*, 11 *aromatic*, 3 admixed), 9 *O. nivara*, 28 *O. rufipogon* accessions, one *O. meridionalis*, one *O. officianialis*, and one *O. punctata* (see Materials and Methods for details on SNP selection criteria).

**Fig. 1:**
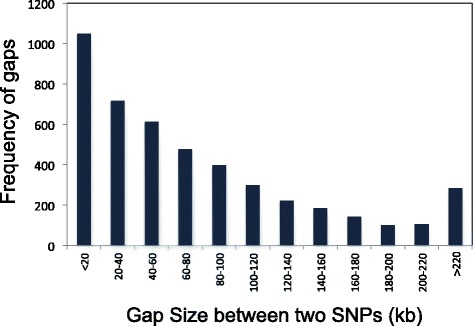
Distribution of SNP distance to its neighboring SNP. More than 50% of SNPs are within 60 kb from a neighboring SNP. The average spacing between SNPs is 79 kb. About 10% of SNPs are >220 kb to the neighboring SNP

Of the 6000 SNPs included in the initial design, 5274 genome-wide SNPs passed Illumina’s production quality control, out of which 1695 SNP markers localized within MSUv7 gene models (Additional file 1: Fig. S1). The average gap between two adjacent SNPs was 79 kb, and more than 50% of SNPs were located within 60 kb of their closest neighbor (Fig. 1). For routine genotyping work at the Genotyping Services Lab at IRRI, a set of 4606 high quality SNPs was used, after filtering SNPs with (a) more than two alleles, (b) duplication of flanking sequences, and (c) high rates of missing data or “no calls” in the targeted *O. sativa* populations. A subset of 4940 SNPs providing high quality data were routinely called at Cornell University, with the higher number most likely due to the inclusion of several *O. rufipogon* and *O. meridionalis* accessions in the analysis.

### Polymorphism rates across pairwise combinations

**Fig. 2:**
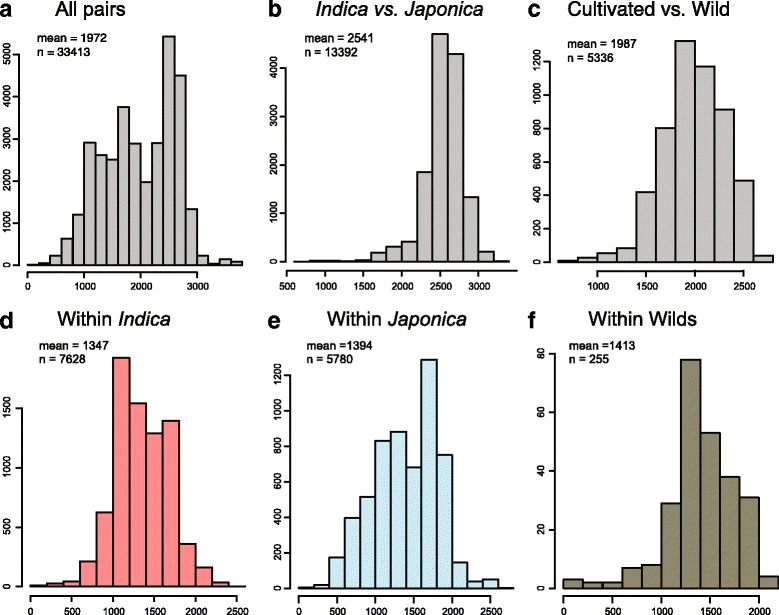
Distribution of the number of polymorphic markers found in pairwise comparisons across diverse germplasm. The number of polymorphic SNPs/pairwise comparison is shown along the x-axis and its count along the y-axis. Panels display the following groups: **a** All pairwise comparisons (*n* = 33,413), **b** *Indica* vs. *Japonica* accessions (*n* = 13,392), **c** Cultivated vs. wild accessions (*n* = 5336), **d** *Indica* vs. *Indica* accessions (*n* = 7628), **e** *Japonica* vs. *Japonica* accessions (*n* = 5780), and **f** Wild vs. wild accessions (*n* = 255). The mean polymorphic SNPs/pairwise comparison are: 1972 (all pairs), 2541 (*Indica* vs. *Japonica*), 1987 (cultivated vs. wild), 1347 (within *Indica*), 1394 (within *Japonica*), 1413 (within wilds)

Pairwise comparisons of polymorphic SNPs between pairs of rice accessions from the same subgroup showed an average of 1347 well-distributed SNPs in *indica*, 1394 SNPs in *japonica*, and 1413 SNPs in wild populations. In contrast, an average of 2541 SNPs were detected between *indica* vs. *japonica* varieties, ~2500 SNPs between *indica* x *aromatic* varieties, ~1500 SNPs between *indica* and genetically divergent *aus* varieties, and an average of 1987 SNPs between either *indica* or *japonica* and the wild accessions evaluated in this study (Fig. 2).

**Fig. 3:**
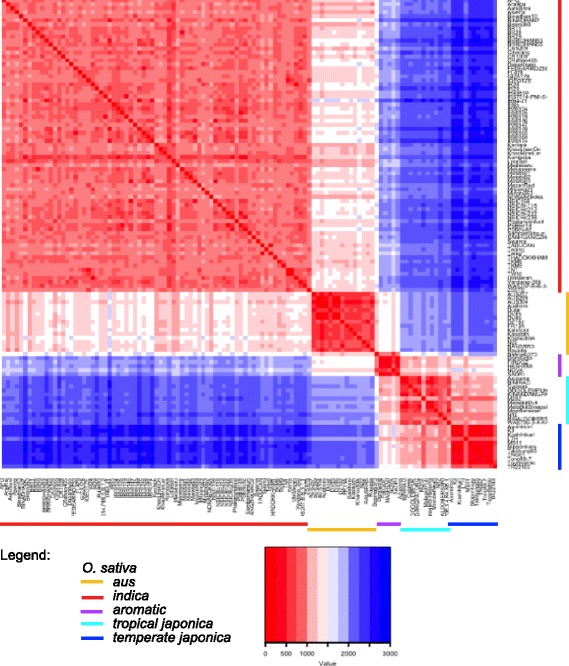
Distribution of the number of polymorphic markers found in pairwise comparisons across the five rice subgroups: *indica, aus, aromatic, temperate japonica* and *tropical japonica*. The number of polymorphic SNPs is indicated in the heatmap index

While the average number of polymorphic markers across diverse germplasm was quite high, polymorphism rates decrease with more closely related germplasm (Fig. 3). For example, *indica* breeding lines at IRRI were often distinguished by only 500 SNPs, and polymorphism rates between two US breeding lines averaged 668 for long grain varieties and 450 SNPs for medium grain varieties (Additional file 2: Fig. S2). Nonetheless, the long and medium grain market classes could still be distinguished using the C6AIR. As can be seen by the number of polymorphic SNPs detected by C6AIR in 18 bi-parental populations commonly used for mapping in rice (Table 1), the pairwise polymorphism rate is more than sufficient for QTL mapping and diversity analyses for all but the most closely related accessions (such as *temperate japonica* X *temperate japonica*).

**Table 1:** Number of polymorphic SNPs detected by C6AIR in 18 bi-parental populations commonly used for mapping in rice. On average a 1 Mb region contains around 3–6 SNPs

Parent1	Subgroup - Parent1	Parent2	Subgroup - Parent2	Number of polymorphic markers between the two parents using C6AIR
DV85	*aus*	IR24	*indica*	1760
DJ123	*aus*	IR64	*indica*	1051
N22	*aus*	IR64	*indica*	1173
N22	*aus*	Swarna	*indica*	1017
Kasalath	*aus*	Nipponbare	*temperate japonica*	1555
DJ123	*aus*	Nipponbare	*temperate japonica*	1506
FR13A	*aus*	M202	*tropical japonica*	1636
Khao Hlan On	*indica*	IR64	*indica*	810
93–11	*indica*	Nipponbare	*temperate japonica*	1742
IR64	*indica*	Azucena	*tropical japonica*	1760
Teqing	*indica*	Lemont	*tropical japonica*	1713
Minghui 63	*indica*	Azucena	*tropical japonica*	1578
Jasmine85	*indica*	Lemont	*tropical japonica*	1629
IR64	*indica*	Jefferson	*tropical japonica*	1718
Nipponbare	*temperate japonica*	FR13A	*aus*	1627
Nipponbare	*temperate japonica*	IR64	*indica*	1982
Geumobyeo	*temperate japonica*	Moroberekan	*tropical japonica*	866
Kinandang Patong	*tropical japonica*	IR64	*indica*	1889

### Applications of the Cornell 6 K rice array in genetics and breeding programs

The C6AIR has been used extensively in two institutions, Cornell University and IRRI. Cornell uses the C6AIR chip for pre-breeding, to develop introgression lines using the rice wild relatives *O. rufipogon* and *O. meridionalis,* for QTL mapping, and to genotype elite material used in US breeding programs, mainly *tropical japonica* varieties. On the other hand, IRRI’s focus is mainly on *indica* varieties, though researchers in both institutions utilize all five *O. sativa* subgroups for genetics and breeding applications. Recent publications also highlight the utility of the Cornell 6 K rice chip for QTL analysis of heat, salinity and flooding tolerance (Ye et al. 2015; Gimhani et al. 2016; Gonzaga et al. 2017; Singh et al. 2017). Three additional applications are presented below: diversity analysis, tracking introgressions, and developing pre-breeding materials.

### Diversity and genetic analysis

**Fig. 4:**
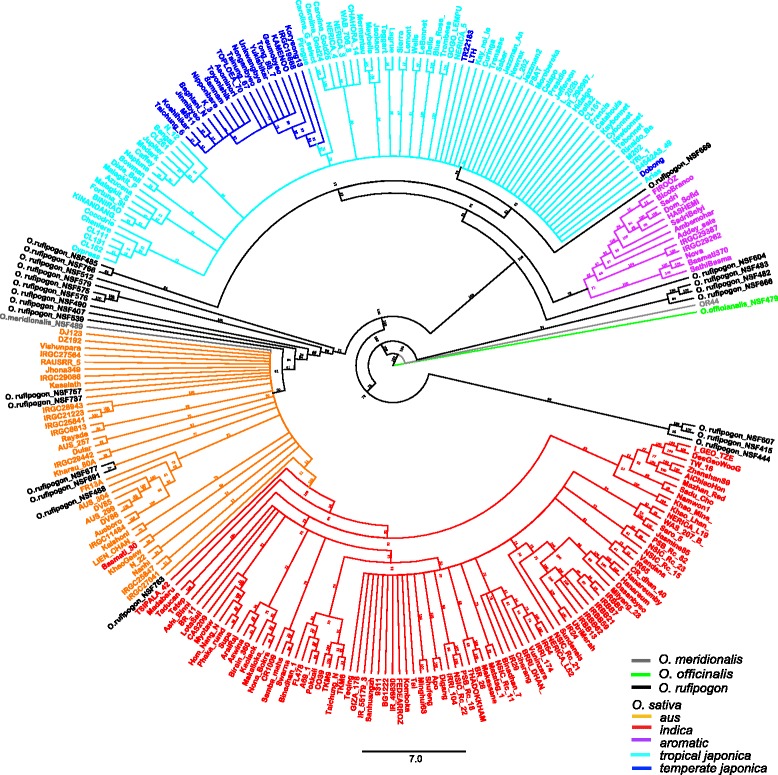
Distance tree constructed using the Neighbor-Joining method based on 232 *Oryza sativa*, 23 *Oryza rufipogon*, 2 *O. meriodionalis* (AA genome) and 1 *Oryza officinalis* (outgroup) accessions.A total of 4940 SNP data points from the C6AIR was used for the analysis and the number at the nodes indicate boot- strap value (100 replicates). The colors in the tree correspond to species and subpopulations; *Oryza officinalis* indicated in light green; *O. meriodionalis* in dark green; *Oryza rufipogon* in black, and the five subpopulations of *Oryza sativa* are in orange (*aus*), red (*indica*), purple (*aromatic or Group V*), cyan (*tropical japonica*) and blue (*temperate japonica*)

Using the C6AIR, a set of diverse germplasm consisting of 232 *O. sativa*, 23 *O. rufipogon* (AA genome), 2 *O. meriodionalis* (AA genome) and 1 *Oryza officinalis* (CC genome) accessions were genotyped (Additional file 3: Table S1). Diversity analysis using the neighbor-joining method defined the five *O. sativa* subgroups: *indica*, *aus*, *aromatic*, *temperate japonica* and *tropical japonica*, though the two *japonica* subgroups were poorly discriminated (Fig. 4). Consistent with previous analyses, *aromatic* varieties are closely related to *japonica* varieties (Garris et al. 2005; Zhao et al. 2010). We also genotyped *O. rufipogon* accessions which were distributed throughout the tree, many with long branch lengths (Fig. 4). The result confirmed the ability of the C6AIR to detect diversity within *O. rufipogon*.

Further analysis using Principal Component Analysis (PCA) of 232 *O. sativa* accessions also showed that genotype data from the C6AIR were able to distinguish the five major subgroups of *O. sativa* (Additional file 4: Fig. S3). The first PC (PC1) separated *indica* and *aus* varieties from *japonica* and *aromatic* varieties. The second PC (PC2) was able to distinguish *tropical japonica*, *temperate japonica* and *aromatic* varieties. Finally, the third PC (PC3) distinguished *indica* from *aus* varieties.

### Tracking introgression of target QTLs during marker-assisted backcrossing

In marker assisted backcrossing (MABC), it is desirable to be able to rapidly introgress chromosomal regions containing a gene or QTL of interest, while at the same time selecting for small size of a target introgression to avoid linkage drag. We highlight two examples of tracking introgressions using C6AIR: one for the cloned and well-characterized *SUB1* gene and another for the *Xa7* gene*. SUB1* confers submergence tolerance for up to 14 days at the vegetative stage, and the gene responsible for the phenotype has been cloned and characterized (Xu et al. 2006; Septiningsih et al. 2009). *Xa7* is a bacterial leaf blight resistance gene from the *aus* variety DV85. It is effective at high temperature, which makes it a promising gene for bacterial leaf blight resistance in areas affected by high temperature (Webb et al. 2009). *Xa7* is located on chromosome 6 between 27.9–28.0 Mb, but the candidate gene has not yet been cloned.

**Fig. 5:**
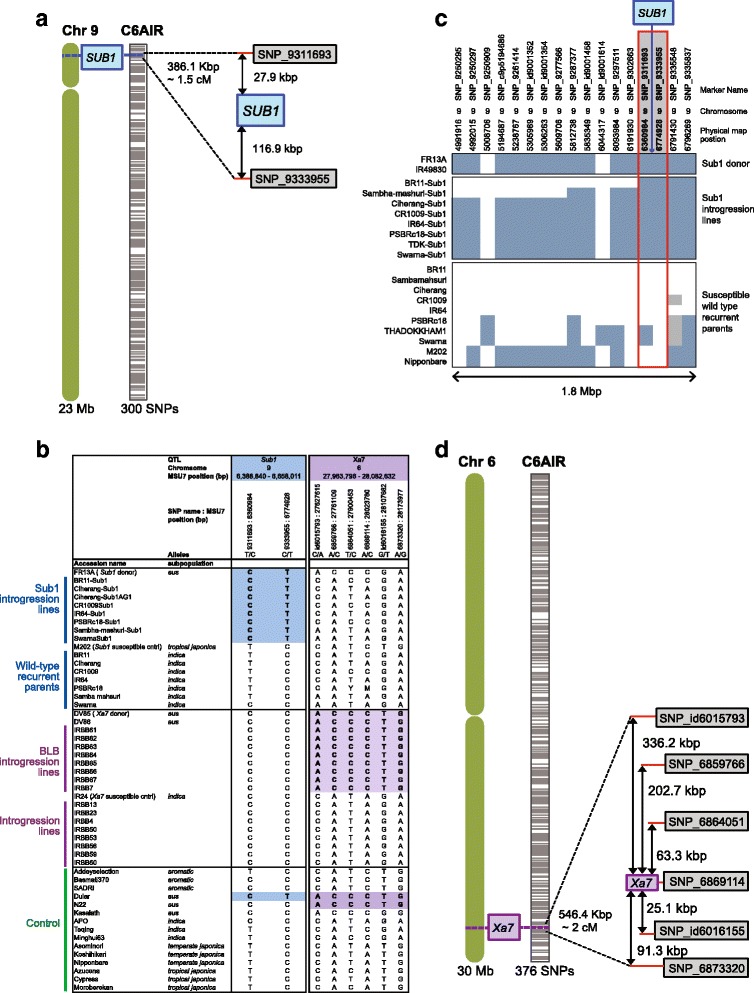
Tracking QTL introgressions on chromosomes 6 and 9 of rice using the C6AIR. **a** QTL for *Sub1* was previously mapped to chromosome 9 at 6,388,840–6,658,011 bp (MSUv7), (Xu et al. 2006; Septiningsih et al. 2009). Two C6AIR markers that localize close to *Sub1* (~1.5 cM region) can be used to track the presence or absence of the QTL for development of *Sub1* introgression lines (blue box = *Sub1*, gray boxes = nearby markers). **b** Table shows genotypes of *Xa7* and *Sub1* predictive markers in popular rice varieties (control) and their derived introgression lines that carry *Xa7* and *Sub1*. **c** C6AIR genotype calls around the *Sub1* QTL region (~1.8 Mb) *Sub1* donors, Sub1-introgression lines and wild-type recurrent parents. Introgression size of varieties carrying a functional *SUB1A* gene vary, i.e. BR11-*Sub*1 and Sambha-Mahsuri-*Sub*1 have the smallest introgressed regions among the *SUB1* varieties (blue box = Sub1 genotype, white box = wild type genotype, gray boxes = missing data). **d** QTL for *Xa7* is located on chromosome 6 at MSU7 position 27,963,796–28,082,632 bp (Chen et al. 2008). Five C6AIR markers localize close to *Xa7* and one SNP is within the *Xa7* region. These markers can be used to track the presence or absence of the QTL for development of *Xa7* introgression lines (purple box = *Xa7*, gray boxes = nearby markers)

The *SUB1* region contains three *SUB1* genes (*SUB1A*, *SUB1B*, *SUB1C*), located between 6.3–6.7 Mb on chromosome 9 (Fig. 5a). *SUB1C* (LOC_Os09g11460) and *SUB1B* (LOC_Os09g11480) are present in Nipponbare, but *SUB1A*, which is responsible for submergence tolerance, is missing from the reference genome (Xu et al. 2006). Based on a comparison of *SUB1* and non-*SUB1* varieties, two SNPs on the C6AIR located at 6,360,984 bp and 6,774,928 bp are able to clearly distinguish varieties that do or do not carry *SUB1A* (Fig. 5b). In addition, varieties with different size introgressions can be distinguished; BR11-*Sub*1 and Sambha-Mahsuri-*Sub*1 have smaller introgressed regions around the *SUB1* locus than other varieties examined (Fig. 5c).

The *Xa7* introgression from DV85 could be readily tracked using the C6AIR in backcross lines with the recurrent parent, IR24 (i.e., the IRBB isolines). The introgression from DV85 into IR24 spanned between 26.3 Mb to 28.3 Mb (Fig. 5d). Lines carrying the resistant *Xa7* allele could be distinguished from susceptible lines by 16 SNPs that mapped within the introgressed region, including one SNP that is located in the previously reported *Xa7* QTL region (Chen et al. 2008). These SNPs, although not functional, are in perfect linkage disequilibrium (LD) with the causal polymorphism in this material, and therefore can be utilized to identify varieties carrying the favorable allele. Trait-specific haplotypes defined by SNP markers discovered using the C6AIR can also be readily converted to other genotyping platforms, such as KASP, Taqman, or Fluidigm, which may be more cost effective for forward selection in breeding programs that target a few, large effect loci.

In addition to foreground selection, the C6AIR is ideally suited for comprehensive background selection in a marker assisted backcrossing (MABC) scheme, where the objective is to rapidly return the genetic background to the recurrent parent type. For example, in an MABC program aiming to transfer the *Pup1* QTL for phosphorous uptake from IR74-*Pup1* to TR22183, BC_2_F_1_ individuals having the target introgression were screened with the C6AIR to identify the lines with the fewest donor introgressions in the background (Additional file 5: Fig. S4). Combining low-cost foreground and recombinant selection (using KASP markers) with high-resolution background selection (using the C6AIR) provides a powerful strategy for rapid and precise MABC transfer of introgressions into elite genetic backgrounds.

### Developing sets of CSSLs between *O. sativa* and *O. rufipogon*

Six rice CSSL libraries were developed from crosses between three *O. rufipogon* accessions (‘Khao Pa’, W1944, IRGC105567) and two elite recurrent parents, the *indica* IR64 and *tropical japonica* Cybonnet. Genotyping with the C6AIR provided between 1311 to 1952 polymorphic genome-wide SNPs per library, and was used in each backcross generation to identify lines that carried the *O. rufipogon* introgression of interest, to precisely delimit the size of the target introgression, and to select against unwanted donor introgressions in the background. Although CSSL development was initiated using 384-SNP GoldenGate assays, the C6AIR proved to be much more efficient and informative for CSSL development than the lower resolution assays that preceded it.

**Fig. 6:**
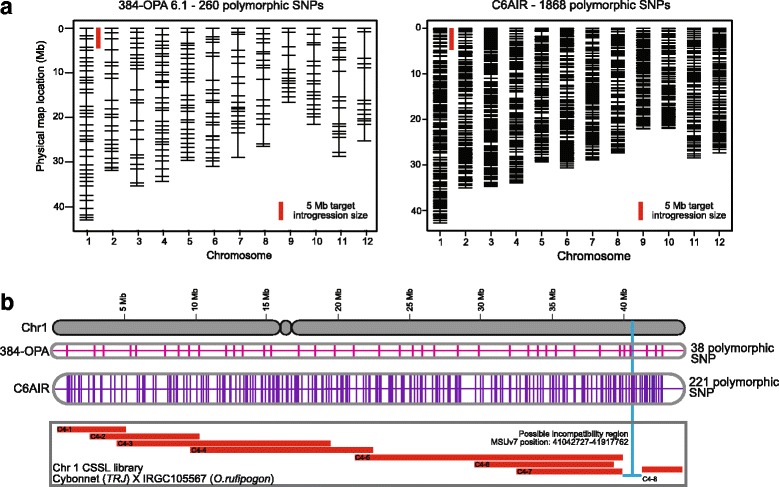
Comparison of the use of 384-SNP Golden Gate assay (OPA 6.1) and C6AIR genotyping platforms for foreground and background selection. CSSLs were developed between the elite *tropical japonica* variety Cybonnet as the recurrent parent, and *O. rufipogon*, IRGC105567, as the donor parent. **a** Distribution of 260 informative SNPs across 12 rice chromosomes detected using the 384-SNP assay (OPA 6.1) (left) and 1868 polymorphic SNPs using the C6AIR (right). The red bar indicates 5 Mb target introgression from the donor parent in the background of Cybonnet. Using the 384-OPA, an average 5 Mb region harbors ~1–3 informative SNPs; using the C6AIR, a 5 Mb region harbors ~5–8 informative SNPs. **b** Graphical representation of CSSL selection targeting overlapping introgressions on Chromosome 1 in the Cybonnet X IRGC105567 library using the 384-OPA 6.1 platform (38 polymorphic SNPs detected) (pink), and the C6AIR (221 polymorphic SNPs) (purple). Blue line indicates a region of putative incompatibility or sterility identified in this population

A comparison between 384-SNP OPA 6.1 and C6AIR genotyping platform was done by using the informative SNPs for foreground and background selection during the development of CSSLs between Cybonnet and *O.rufipogon* parent IRGC105567 (NSF490) (Fig. 6). The low resolution 384-OPA identified 260 polymorphic SNPs sparsely distributed across the 12 rice chromosomes whereas the C6AIR detected 1868 well-distributed polymorphic SNPs (Fig. 6a). Each line was selected to contain a target 5–7 Mb introgression; using the 384-OPA, a 5 Mb region had an average of 1–3 informative SNPs while the C6AIR detected 5–8 informative SNPs across the same size region, thus providing a better approximation of introgression size.

Eight CSSLs provided coverage of chromosome 1 in the Cybonnet X NSF490 population (C4–1 to C4–8), each line carrying a homozygous donor segment in the background of the recurrent parent (Fig. 6b). During selection of lines representing C4–7 and C4–8, several segregating plants were found to have unfilled panicles. Genotyping analysis of the lines that produced seeds showed them to be either heterozygous towards the end of Chr 1 (38–42 Mb) or homozygous for the recurrent parent. To narrow down this putative incompatibility or sterility region, polymorphic SNPs between the two platforms were compared alongside the Chr1-CSSLs. The 384-OPA 6.1 detected 38 polymorphic SNPs on chromosome 1 and identified the putative incompatibility region at a resolution of 4.38 Mb (~17 cM) (between positions 38.58–42.96 Mb). Using the C6AIR, there were 221 informative SNPs on chr1, and the region of interest was narrowed down to 0.875 Mb (~3 cM) (between positions 41.04–41.91 Mb) (Fig. 6b).

### Design and characteristics of the improved C7AIR

To further improve the C6AIR, a second-generation Infinium rice array was recently developed by eliminating SNPs that performed poorly on the 6 K array and increasing the number of attempted bead types. This new 7 K–SNP array, referred to commercially as the Cornell-IR LD Rice Array, and by the user community as the Cornell_7K_Array_Infinium_Rice (C7AIR), provides continuity with the C6AIR, while increasing the number of high quality SNP loci that can be interrogated. The C7AIR includes 4007 SNPs from the 6 K array, 2056 SNPs from the 700,000-SNP HDRA selected to be informative for US rice germplasm, 910 SNPs from the 384-SNP GoldenGate sets (OPA2.1, 3.1, 4.0, 5.0, 6.2 and 7.0), 189 SNPs from the 44 K array selected to be informative for US rice germplasm, and 21 gene-based SNPs from IRRI. SNPs on the C7AIR are expected to be informative for detecting genome-wide polymorphism between individuals within the *indica*, *aus*, and *tropical japonica* subpopulations, between any pairwise combination of accessions from the *indica*, *aus*, *tropical japonica*, *temperate japonica*, or *aromatic* (Group V) subpopulations, and between *O. sativa* and *O. rufipogon*. The array is expected to be moderately informative for detecting polymorphism within the *aromatic* (Group V) subpopulation, and least informative for detecting polymorphism within the *temperate japonica* subpopulation. Trait-specific markers include diagnostic SNPs for the SUB1A gene, grain quality characteristics, and loci for resistance to bacterial leaf blight, blast, brown planthopper, and tungro to enhance C7AIR utility for foreground selection and QTL profiling. The C7AIR is being beta-tested at this time and will be manufactured by Illumina as a consortium array for future use in rice genetics and breeding worldwide.

## Conclusions

The C6AIR has proven to be an effective genotyping system for rice diversity analysis, QTL mapping, tracking introgressions, genetic stock development, and fingerprinting studies at both Cornell University and at the Genotyping Services Lab at IRRI over the last 4 years. Over 40,000 samples were run successfully, providing genotyping data for a large number of genetics, breeding and impact assessment projects of importance to people throughout the world. Arrays such as the C6AIR provide a relatively small but sufficient number of SNPs so that data can be handled without massive bioinformatics pipelines, while at the same time providing high enough resolution for most genetics and breeding applications. The SNPs on the C6AIR consistently have high call rates, including for heterozygotes, facilitating data management and integration of genotyping data across runs and populations. Because the C6AIR can readily distinguish the five major subgroups of *O. sativa* as well as *O. rufipogon*, it is especially useful for fingerprinting studies and, as summarized in this paper, finds a broad range of applications in rice breeding programs. The improved second generation C7AIR offers all of the benefits of the 6 K array with additional high quality SNP markers. These resources provide the rice community with rapid, cost-effective tools for low-density, genome-wide SNP genotyping across a wide range of rice germplasm, and with the potential to dramatically increase resolution via imputation when integrated with other publicly available high-density rice genome datasets.

## Methods

### Plant materials

Rice accessions used in this study are listed in Additional file 3: Table S1. Genomic DNA (gDNA) was extracted from leaf tissue of single plants using the CTAB, Qiagen or SBEadex methods (Fulton et al. 1995). The quality of DNA was checked visually on 1% agarose gels, and the quantity was checked using a Nano-Drop spectrophotometer (read at 260/280 nm) and/or a Qubit 2.0 Fluorometer. For target probe preparation, 5 μl of gDNA was used. The concentration of each DNA sample was adjusted to 50–100 ng/μl.

### Design of the C6AIR and the C7AIR

The custom-designed Infinium iSelect C6AIR consisted of 6000 attempted bead types, including 1571 SNP markers from legacy BeadXpress 384-SNP sets (Thomson et al. 2012) and 4429 SNPs selected from re-sequencing data available in the McCouch lab, as described in McCouch et al. (2016). Of these, 2000 SNPs were selected that segregated at mid frequencies within each of the 5 subpopulations of *O. sativa (*400 non-overlapping SNPs/subpopulation), and 2429 SNPs were selected to be polymorphic for specific bi-parental cross combinations (including the *O. sativa* and *O. rufipogon* parents of the targeted CSSL populations). A larger candidate set of 800,468 SNPs were initially scored by the Illumina Assay Design Tool (ADT). After scoring the candidate set of SNPs, the subsequent selection of markers was done through several levels of filtering, including basic filtering: i) there were no SNPs within +/− 10 bp of target on either side; ii) no SNPs with minor homozygote count of more than 4 within +/− 35 bp occurring on both sides; iii) INDELS were removed; iv) SNPs with repetitive sequence and low minor allele frequencies in the set of 128 re-sequenced genomes were omitted; and specific filtering: the remaining SNPs were then screened for segregation properties of specific cross combinations, and for segregation within each of the 5 major subpopulations at minor allele frequency > 20% and observation rate within subpopulation >80%. The union set of SNPs, which satisfy either the subpopulation allele frequency criteria or cross segregation criteria were those that became candidates for the array. The final filtering was done to optimize genome spacing and polymorphism detection within and between species and subpopulations. The minimum resolution was at least 1 informative SNP per Mb for the intra- and interspecific bi-parental cross combinations used during the design phase. The SNP information is provided in Additional file 6: Table S2.

The C7AIR, referred to by Illumina as the Cornell-IR LD Rice Array, represents an improved version of the C6AIR. It was designed by eliminating SNPs that performed poorly on the C6AIR (Additional file 7: Fig. S5) and selecting high quality SNPs from arrays previously designed in the McCouch lab (Zhao et al. 2010; Zhao et al. 2011; McCouch et al. 2016) to increase the number of SNPs that would be informative for interrogating elite *tropical japonica* breeding material.

### Genotyping and SNP allele calling

DNA amplification was performed following the manufacturer’s protocol. PCR products were hybridized to the Infinium II BeadChip and fluorescently stained following the manufacturer’s protocol. The fluorescence intensity of the beadchip was scanned using an Illumina BeadArray Reader. The raw data of the scanned Infinium 6 K BeadChips from the BeadArray Reader were decoded to generate SNP data using the GenomeStudio Software. At Cornell a subset of 4940 SNPs were filtered based on call rate (80%) from 5274 SNPs that passed Illumina’s QC. Whereas, at IRRI a customized cluster file was used to generate the forward strand SNP data. The cluster file used at IRRI consists of 4606 high quality SNP markers, which had undergone stringent filtering. Representative samples coming from the 5 subgroups of cultivated rice (ie. *indica, tropical japonica*, *temperate japonica*, *aus* and *aromatic*) were used to determine the efficiency of the filtered markers in segregating the aforementioned groups. Final SNP data were merged with SNP map (Nipponbare MSU7) information and encoded with the physical position and chromosome number of the 4606 SNP markers (Additional file 6: Table S2).

### Tree construction

To resolve the genetic relationships among the different rice subgroups, 258 rice accessions were analyzed. A total of 5274 SNPs were filtered in TASSEL GUI 5.2.37 using minor allele count (MAC) = 5, call rate = 0.8, resulting in 4940 filtered SNPs that were aligned and used for tree construction using the Geneious Tree Builder. The tree was constructed using the Tamura-Nei genetic distance model, the Neighbor-Joining tree building method, No Outgroup, the Bootstrap Resampling method; and Number of replicates = 100. The tree was visualized using Geneious 8.1.6 to generate a circular cladogram.

## Additional files

Additional file 1: Fig. S1. Genome-wide marker coverage. a) Distribution of 5,274 markers successfully converted from C6AIR, and b) distribution of 1,695 markers that localize within MSUv7 gene models (PDF 291 kb)

Additional file 2: Fig. S2. Distribution of the number of polymorphic markers found in pairwise comparisons across US rice germplasm (PDF 882 kb)

Additional file 3: Table S1. Germplasm information (PDF 96 kb)

Additional file 4: Fig. S3. Principal Components Analysis of diverse cultivated Asian rice accessions: PCA of 232 *O. sativa* accessions (PDF 353 kb)

Additional file 5: Fig. S4. Use of C6AIR for background selection during TR22183-*Pup*1 development (PDF 476 kb)

Additional file 6: Table S2. Information on the 6,000 SNPs used for the C6AIR design (CSV 1977 kb)

Additional file 7: Fig. S5. Distribution of the frequency of “no call” (NC) alleles in (a) cultivated and (b) wild accessions using the Cornell 6 k Array Infinium Rice (PDF 1061 kb)
