# Supplementary material for: Large-scale deployment of a rice 6 K SNP array for genetics and breeding applications
Source: Rice (N Y). 2017 Aug 30;10:40. doi: 10.1186/s12284-017-0181-2 (PMC5577349; doi:10.1186/s12284-017-0181-2)
Supplement: Supplementary file 1 — Genome-wide marker coverage. a) Distribution of 5,274 markers successfully converted from C6AIR, and b) distribution of 1,695 markers that localize within MSUv7 gene models (PDF 291 kb) [file 12284_2017_181_MOESM1_ESM.pdf]

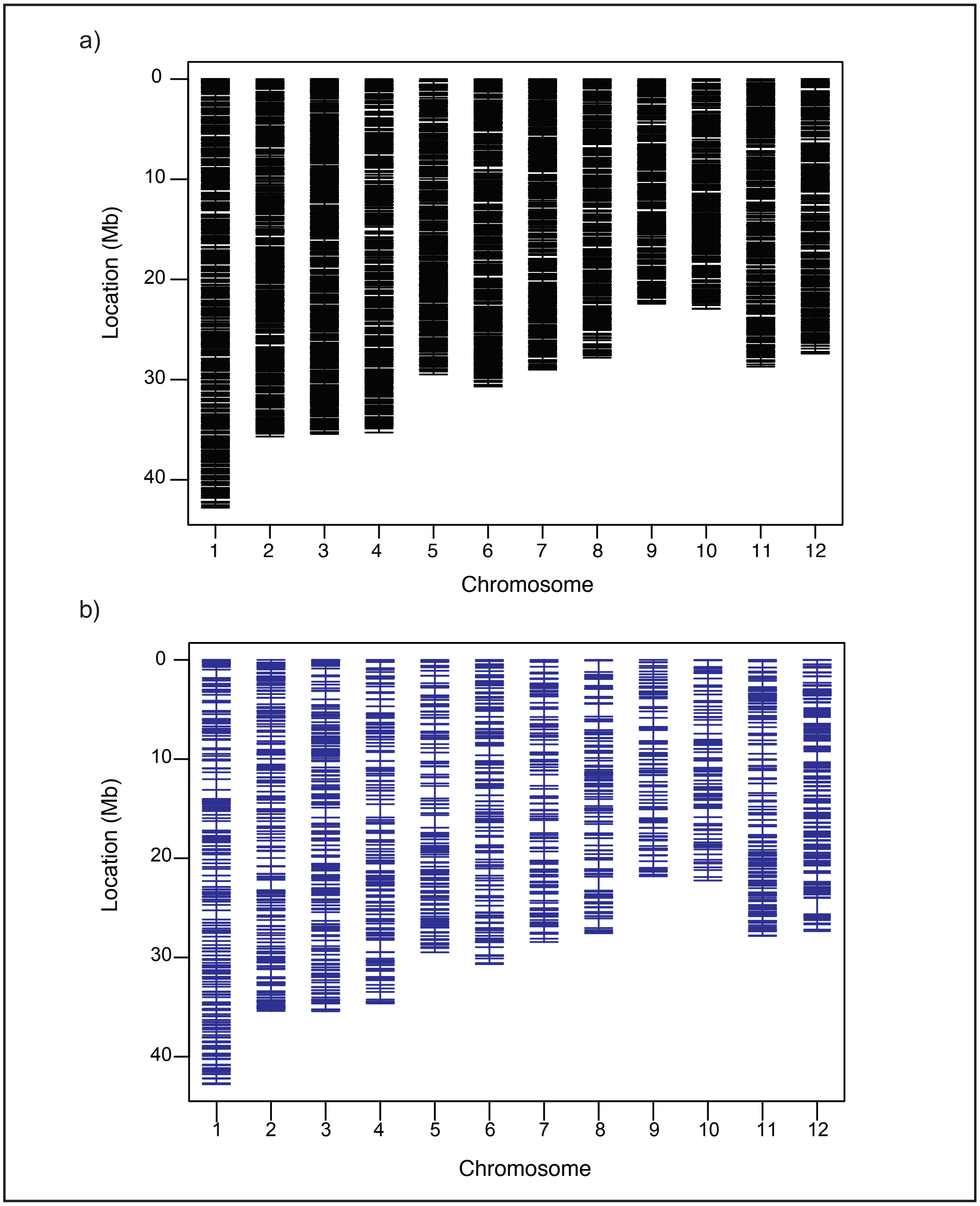

Figure S1. Genome-wide marker coverage. a) Distribution of 5,274 markers successfully converted from C6AIR, and b) distribution of 1,695 markers that localize within an MSUv7 gene models.
