# Supplementary material for: Large-scale deployment of a rice 6 K SNP array for genetics and breeding applications
Source: Rice (N Y). 2017 Aug 30;10:40. doi: 10.1186/s12284-017-0181-2 (PMC5577349; doi:10.1186/s12284-017-0181-2)
Supplement: Supplementary file 2 — Distribution of the number of polymorphic markers found in pairwise comparisons across US rice germplasm (PDF 882 kb) [file 12284_2017_181_MOESM2_ESM.pdf]

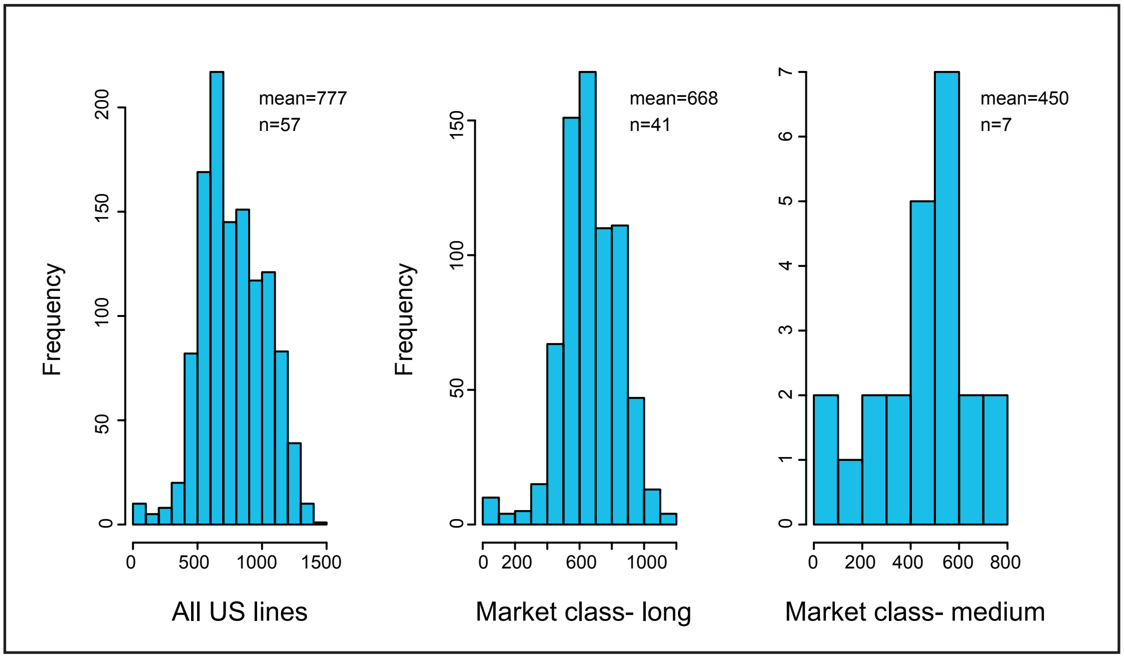

Figure S2. Distribution of the number of polymorphic markers found in pairwise comparisons across US rice germplasm. Number of polymorphic SNPs/pairwise comparison shown along the x-axis and its count along the y-axis. Panels display the following groups: a) All US lines pairwise comparisons (n= 57), b) Market class long grain (n=41), c) Market class medium grain (n=7).
