# Supplementary material for: Large-scale deployment of a rice 6 K SNP array for genetics and breeding applications
Source: Rice (N Y). 2017 Aug 30;10:40. doi: 10.1186/s12284-017-0181-2 (PMC5577349; doi:10.1186/s12284-017-0181-2)
Supplement: Supplementary file 3 — Germplasm information (PDF 96 kb) [file 12284_2017_181_MOESM3_ESM.pdf]

Table S1. Germplasm information

A. *O. sativa* accessions

S. No	IRGC ID	NSF ID	GSOR ID	Accession name	Species	Subpopulation	Country	Institute
1				Ambemohar	*O. sativa*	*aromatic*	India	CU
2	177			Addey selection	O. sativa	*aromatic*	India	IRRI
3	121899			Basmati_370	*O. sativa*	*aromatic*	India	CU
4	117658	NSFTV16	GSOR301014	Bico_Branco	*O. sativa*	*aromatic*	Brazil	CU
5				Dom Sofid	O. sativa	*aromatic*	Iran	IRRI
6				Firooz	O. sativa	*aromatic*	Iran	IRRI
7				Hashemi	O. sativa	*aromatic*	Iran	IRRI
8	29262			IRGC29262	O. sativa	*aromatic*	Bangladesh	IRRI
9	29387			IRGC29387	O. sativa	*aromatic*	Bangladesh	IRRI
10				Nova	O. sativa	*aromatic*	United States	IRRI
11				Sadri	O. sativa	*aromatic*	Iran	IRRI
12	117872	NSFTV221	GSOR 301212	Sadri_Belyi	*O. sativa*	*aromatic*	Azerbaijan	CU
13	117878		GSOR 301356	Sathi_Basmati	*O. sativa*	*aromatic*	Pakistan	CU
14				Aus 257	O. sativa	*aus*	Bangladesh	IRRI
15				Aus 299	O. sativa	*aus*	Bangladesh	IRRI
16				Aus 304	O. sativa	*aus*	Bangladesh	IRRI
17	36983			Ausboro	O. sativa	*aus*	Bangladesh	IRRI
18	117711	NSFTV317	GSOR 301307	DJ123	*O. sativa*	*aus*	Bangladesh	CU
19				Dular	O. sativa	*aus*	Bangladesh/India/Pakistan	IRRI
20				DV85	O. sativa	*aus*	Bangladesh	IRRI
21				DV86	O. sativa	*aus*	Bangladesh	IRRI
22				DZ192	O. sativa	*aus*	Bangladesh	IRRI
23				FR13A	O. sativa	*aus*	India	IRRI
24	11484			IRGC11484	O. sativa	*aus*	India	IRRI
25	21223			IRGC21223	O. sativa	*aus*	India	IRRI
26	25841			IRGC25841	O. sativa	*aus*	Bangladesh	IRRI
27	25847			IRGC25847	O. sativa	*aus*	Bangladesh	IRRI
28	27041			IRGC27041	O. sativa	*aus*	Indonesia	IRRI
29	27564			IRGC27564	O. sativa	*aus*	Bangladesh	IRRI
30	28943			IRGC28943	O. sativa	*aus*	Bangladesh	IRRI
31	29086			IRGC29086	O. sativa	*aus*	Bangladesh	IRRI
32	29442			IRGC29442	O. sativa	*aus*	Bangladesh	IRRI
33	6613			IRGC6613	O. sativa	*aus*	Bangladesh	IRRI
34	117769	NSFTV78	GSOR 301071	Jhona349	*O. sativa*	*aus*	India	CU
35				Kal Shoni	O. sativa	*aus*	Bangladesh	IRRI
36	117617	NSFTV85	GSOR 301077	Kasalath	*O. sativa*	*aus*	India	CU
37	117781	NSFTV88	GSOR 301080	Khao_Gaew	*O. sativa*	*aus*	Thailand	CU
38	28017			Kharsu 80A	O. sativa	*aus*	Pakistan	IRRI
39				Lien Chan Sha Pu Tsan	O. sativa	*aus*	China	IRRI
40				N 22	O. sativa	*aus*	India	IRRI
41	74769			Nanhi	O. sativa	*aus*	India	IRRI
42				RAUSRR 5	O. sativa	*aus*	India	IRRI
43				Rayada	O. sativa	*aus*	Bangladesh	IRRI
44	-	NSFTV398	GSOR301399*	9311	*O. sativa*	*indica*	China	CU
45				A69-1	O. sativa	*indica*	Philippines	IRRI
46	117636	NSFTV3	GSOR 301003	Ai-Chiao-Hong	*O. sativa*	*indica*	China	CU
47				Apo	O. sativa	*indica*	Philippines	IRRI
48				Arai Raj	O. sativa	*indica*	Bangladesh	IRRI
49	77216			Ashi Binni	O. sativa	*indica*	Bangladesh	IRRI
50				Aswina	O. sativa	*indica*	Bangladesh	IRRI
51	83309			Basmati 306	O. sativa	*indica*	India	IRRI
52				BG 1222	O. sativa	*indica*	Sri Lanka	IRRI
53				BINA Dhan-10	O. sativa	*indica*	Bangladesh	IRRI
54				BINA Dhan-7	O. sativa	*indica*	Bangladesh	IRRI
55	6550			Birain 360	O. sativa	*indica*	Bangladesh	IRRI
56				BR 11	O. sativa	*indica*	Bangladesh	IRRI
57				BR 28	O. sativa	*indica*	Bangladesh	IRRI
58				BRRI DHAN 53	O. sativa	*indica*	Bangladesh	IRRI
59				CAS 209	O. sativa	*indica*	Senegal	IRRI
60				Ciherang	O. sativa	*indica*	Indonesia	IRRI
61	117454		GSOR301524	CO39	*O. sativa*	*indica*	India	CU
62				CR dhan 405	O. sativa	*indica*	Philippines	IRRI
63				CR1009	O. sativa	*indica*	India	IRRI
64				Dasanbyeo	O. sativa	*indica*	South Korea	IRRI
65	117705	NSFTV43	GSOR 301040	Dee_Geo_Woo_Gen	*O. sativa*	*indica*	Taiwan	CU
66				Digang	*O. sativa*	*indica*		CU
67				Fedearroz 50	O. sativa	*indica*	Colombia	IRRI
68				FL478	O. sativa	*indica*	Philippines	IRRI
69				Hanareum	O. sativa	*indica*	South Korea	IRRI
70				Hanareumbyeo	O. sativa	*indica*	South Korea	IRRI
71	5642			Hom Nang Nouane	O. sativa	*indica*	Lao PDR	IRRI
72	120			I Geo Tze	O. sativa	*indica*	Taiwan	IRRI
73				IR 49830	O. sativa	*indica*	Philippines	IRRI

S. No	IRGC ID	NSF ID	GSOR ID	Accession name	Species	Subpopulation	Country	Institute
74				IR 55179-3B-11-3	O. sativa	*indica*	Philippines	IRRI
75				IR24	O. sativa	*indica*	Philippines	IRRI
76				IR29	O. sativa	*indica*	Philippines	IRRI
77	117268	NSFTV612	GSOR301401	IR64	*O. sativa*	*indica*	Philippines	CU
78				IR65	O. sativa	*indica*	Philippines	IRRI
79				IRBB13	O. sativa	*indica*	Philippines	IRRI
80	A7484			IRBB21	O. sativa	*indica*	Philippines	IRRI
81				IRBB5	O. sativa	*indica*	Philippines	IRRI
82				IRBB59	O. sativa	*indica*	Philippines	IRRI
83	115114			IRBB62	O. sativa	*indica*	Philippines	IRRI
84				IRBB7	O. sativa	*indica*	Philippines	IRRI
85				IRRI 104	O. sativa	*indica*	Philippines	IRRI
86				IRRI 174	O. sativa	*indica*	Philippines	IRRI
87	301406	NSFTV620		Jasmine85	*O. sativa*	*indica*	Philippines	CU
88	61381			Kaolack	O. sativa	*indica*	Guinea	IRRI
89	88335			Khao Hlan On	O. sativa	*indica*	Myanmar	IRRI
90	88336			Khao Mine Lar	O. sativa	*indica*	Myanmar	IRRI
91				Komboka	O. sativa	*indica*	Philippines	IRRI
92				Lota Sail	O. sativa	*indica*	Bangladesh	IRRI
93	60184			Ma-Zhan Red	O. sativa	*indica*	China	IRRI
94	15333			Madabaru	O. sativa	*indica*	Sri Lanka	IRRI
95				Makalioka 34	O. sativa	*indica*	Madagascar	IRRI
96				Makassane	O. sativa	*indica*	Philippines	IRRI
97	497625			Matatag 1	O. sativa	*indica*	Philippines	IRRI
98				Milyang 23	O. sativa	*indica*	North/South Korea	IRRI
99				Minghui 63	O. sativa	*indica*	China	IRRI
100	33421			Myotaw	O. sativa	*indica*	Myanmar	IRRI
101				Namwon 1	O. sativa	*indica*	South Korea	IRRI
102				NERICA_L19	O. sativa	*indica*	Benin/Senegal	CU
103				NERICA_L52	O. sativa	*indica*	Benin/Senegal	CU
104				Nona Bokra	O. sativa	*indica*	India	IRRI
105	1329424			NSIC Rc 118	O. sativa	*indica*	Philippines	IRRI
106				NSIC Rc 158	O. sativa	*indica*	Philippines	IRRI
107				NSIC Rc 212	O. sativa	*indica*	Philippines	IRRI
108				NSIC Rc 222	O. sativa	*indica*	Philippines	IRRI
109				NSIC Rc 238	O. sativa	*indica*	Philippines	IRRI
110				Phka Rumduol	O. sativa	*indica*	Cambodia	IRRI
111				Pokkali	O. sativa	*indica*	india, sri lanka	IRRI
112				PSB Rc 18	O. sativa	*indica*	Philippines	IRRI
113				PSB Rc 82	O. sativa	*indica*	Philippines	IRRI
114				Sadu-Cho	O. sativa	*indica*	South Korea	IRRI
115				Samba Mahsuri	O. sativa	*indica*	India	IRRI
116				Sanhuangzhan No 2	O. sativa	*indica*	China	IRRI
117				Saro 5	O. sativa	*indica*	Tanzania	IRRI
118				Sundensis	O. sativa	*indica*	Kazakhstan	IRRI
119				Supa	O. sativa	*indica*	Kenya, Tanzania, Zambia	IRRI
120				Swarna	O. sativa	*indica*	India	IRRI
121				Shufeng	*O. sativa*	*indica*	United States	IRRI
122	117906	NSFTV163	GSOR 301154	Taducan	*O. sativa*	*indica*	Philippines	CU
123				Tai	O. sativa	*indica*	Philippines	IRRI
124				Taichung Native 1	O. sativa	*indica*	Taiwan	IRRI
125	539463			Teqing	O. sativa	*indica*	China	IRRI
126				Tetep	O. sativa	*indica*	India	IRRI
127				THADOKKHAM 1	O. sativa	*indica*	Lao PDR	IRRI
128				TKM 6	O. sativa	*indica*	India	IRRI
129	69338			TKM 9	O. sativa	*indica*	India	IRRI
130				TSIPALA 421	O. sativa	*indica*	Madagascar	IRRI
131				TW 16	O. sativa	*indica*	Philippines	IRRI
132				Utri Merah	O. sativa	*indica*	Indonesia	IRRI
133				Vandana	O. sativa	*indica*	India	IRRI
134	52787			Vishunparag	O. sativa	*aus*	India	IRRI
135				Vuninzara	O. sativa	*indica*	Philippines	IRRI
136				WAS 207-B-B-3-1-1	O. sativa	*indica*	Senegal	IRRI
137				Zhenshan S-97B	O. sativa	*indica*	China	IRRI
138				Giza 178	O. sativa	*indica*	Egypt	IRRI
139				Asominori	O. sativa	*temperate japonica*	South Korea	IRRI
140				Baghlani Nangarhar	O. sativa	*temperate japonica*	Afghanistan	IRRI
141				Dobong	O. sativa	*temperate japonica*	South Korea	IRRI
142	117612	NSFTV56	GSOR 301052	Geumobyeo	*O. sativa*	*temperate japonica*	Republic of Korea	CU
143	19868			IRGC19868	O. sativa	*temperate japonica*	South Korea	IRRI
144				Jinmibyeo	O. sativa	*temperate japonica*	South Korea	IRRI
145				K 3	O. sativa	*temperate japonica*	Cambodia	IRRI
146				Kamenoo	O. sativa	*temperate japonica*	Japan	IRRI
147				Koryeong13	O. sativa	*temperate japonica*	South Korea	IRRI
148				Koshihikari	O. sativa	*temperate japonica*	Japan	IRRI

S. No	IRGC ID	NSF ID	GSOR ID	Accession name	Species	Subpopulation	Country	Institute
149				Lijiangxintuanheigu	O. sativa	*temperate japonica*	China	IRRI
150				MS 11	O. sativa	*temperate japonica*	Philippines	IRRI
151	121592	NSFTV173	GSOR 301164	Nipponbare	*O. sativa*	*temperate japonica*	Japan	CU
152				Nonganbyeo	O. sativa	*temperate japonica*	South Korea	IRRI
153				Samnam	O. sativa	*temperate japonica*	South Korea	IRRI
154				Taichung 65	O. sativa	*temperate japonica*	Taiwan	IRRI
155				Tainung 67	O. sativa	*temperate japonica*	Taiwan	IRRI
156				Tong 88-7	O. sativa	*temperate japonica*	China	IRRI
157				Toploea 70/76	O. sativa	*temperate japonica*	Romania	IRRI
158				Toyonishiki	O. sativa	*temperate japonica*	Japan	IRRI
159				TR22183	O. sativa	*temperate japonica*	China	IRRI
160				Unkwangbyeo	O. sativa	*temperate japonica*	South Korea	IRRI
161				Yukishikari	O. sativa	*temperate japonica*	Japan	CU
162	126381	NSFTV7	GSOR 301007	Arias	*O. sativa*	*tropical japonica*	Indonesia	CU
163	117264	NSFTV174		Azucena	*O. sativa*	*tropical japonica*	Philippines	CU
164		NSFTV622		Bengal	*O. sativa*	*tropical japonica*	United States	CU
165				Binirao	O. sativa	*tropical japonica*	Philippines	IRRI
166		NSFTV182		Blue Rose Supreme	*O. sativa*	*tropical japonica*	United States	CU
167				Bowman	*O. sativa*	*tropical japonica*	United States	CU
168				Caffey	*O. sativa*	*tropical japonica*	United States	CU
169	121302			Caiapo	*O. sativa*	*tropical japonica*	Brazil	CU
170		NSFTV24		Carolina Gold	*O. sativa*	*tropical japonica*	United States	CU
171		NSFTV25		Carolina Gold	*O. sativa*	*tropical japonica*	United States	CU
172				Carolina Gold Select	*O. sativa*	*tropical japonica*	United States	CU
173				Catahoula	*O. sativa*	*tropical japonica*	United States	CU
174				CHAHORA 144	O. sativa	*tropical japonica*	Pakistan	IRRI
175				Cheniere	*O. sativa*	*tropical japonica*	United States	CU
176				CL111	*O. sativa*	*tropical japonica*	United States	CU
177				CL131	*O. sativa*	*tropical japonica*	United States	CU
178				CL151	*O. sativa*	*tropical japonica*	United States	CU
179				CL152	*O. sativa*	*tropical japonica*	United States	CU
180				CL261	*O. sativa*	*tropical japonica*	United States	CU
181		NSFTV396		Cocodrie	*O. sativa*	*tropical japonica*	United States	CU
182				Curinga	*O. sativa*	*tropical japonica*	Brazil	CU
183	117699	NSFTV397	GSOR 301380	Cybonnet	*O. sativa*	*tropical japonica*	United States	CU
184				Cypress	*O. sativa*	*tropical japonica*	United States	CU
185		NSFTV391		Della	*O. sativa*	*tropical japonica*	United States	CU
186				DixieBelle	*O. sativa*	*tropical japonica*	United States	CU
187		NSFTV54		Fortuna (Stuttgart)	*O. sativa*	*tropical japonica*	United States	CU
188		NSFTV632		Francis	*O. sativa*	*tropical japonica*	United States	CU
189				Gogo Lempuk	O. sativa	*tropical japonica*	Indonesia	IRRI
190				Hidalgo	*O. sativa*	*tropical japonica*	United States	CU
191				Jackson	*O. sativa*	*tropical japonica*	United States	CU
192				Jazzman (Anna)	*O. sativa*	*tropical japonica*	United States	CU
193				Jazzman2	*O. sativa*	*tropical japonica*	United States	CU
194	126385	NSFTV628		Jefferson	*O. sativa*	*tropical japonica*	United States	CU
195				Jupiter	*O. sativa*	*tropical japonica*	United States	CU
196	6732			Kay Noi Leuang	O. sativa	*tropical japonica*	Lao PDR	IRRI
197		NSFTV624		Kaybonnet	*O. sativa*	*tropical japonica*	United States	CU
198				Kinandang Patong	O. sativa	*tropical japonica*	Philippines	IRRI
199				L-202	*O. sativa*	*tropical japonica*	United States	CU
200				L-202	*O. sativa*	*tropical japonica*	United States	CU
201		NSFTV621		Lagrue	*O. sativa*	*tropical japonica*	United States	CU
202				Lebonnet	*O. sativa*	*tropical japonica*	United States	CU
203		NSFTV101		Lemont	*O. sativa*	*tropical japonica*	United States	CU
204				M 202	O. sativa	*tropical japonica*	United States	IRRI
205	19451			Malagkit Puti	O. sativa	*tropical japonica*	Philippines	IRRI
206				Malagkit Sinaguing	O. sativa	*tropical japonica*	Philippines	IRRI
207				Maybelle	*O. sativa*	*tropical japonica*	United States	CU
208				Medark	*O. sativa*	*tropical japonica*	United States	CU
209				Mermentau	*O. sativa*	*tropical japonica*	United States	CU
210	117621	NSFTV108		Moroberekan	*O. sativa*	*tropical japonica*	Guinea	CU
211				Neptune	*O. sativa*	*tropical japonica*	United States	CU
212				N 12	O. sativa	*tropical japonica*	India	IRRI
213				NERICA_3	*O. sativa*	*tropical japonica*	Benin/Senegal	CU
214				NERICA_4	*O. sativa*	*tropical japonica*	Benin/Senegal	CU
215				NERICA_5	*O. sativa*	*tropical japonica*	Benin/Senegal	CU
216				Newbonnet	*O. sativa*	*tropical japonica*	United States	CU
217				Newrex	*O. sativa*	*tropical japonica*	United States	CU
218				PI 298967-1	O. sativa	*tropical japonica*	Australia	IRRI
219				Pirogue	*O. sativa*	*tropical/temperate japonica*	United States	IRRI
220				Presidio	*O. sativa*	*tropical japonica*	United States	CU
221				PSAT	*O. sativa*	*tropical japonica*	Thailand	CU
222				Rinaldo Bersani	O. sativa	*tropical japonica*	Turkey, Spain, Italy, France	IRRI
223				S4542A3-49B-2B12	O. sativa	*tropical japonica*	United States	IRRI
224				Saber	*O. sativa*	*tropical japonica*	United States	CU

S. No	IRGC ID	NSF ID	GSOR ID	Accession name	Species	Subpopulation	Country	Institute
225				Sierra	*O. sativa*	*tropical japonica*	United States	CU
226				Taggart	*O. sativa*	*tropical japonica*	United States	CU
227				Tebonnet	*O. sativa*	*tropical japonica*	United States	CU
228	117921	NSFTV165	GSOR 301156	Trembese	*O. sativa*	*tropical japonica*	Indonesia	CU
229				Trenasse	*O. sativa*	*tropical japonica*	United States	CU
230				WAB 706-3-4-K4-KB-1	O. sativa	*tropical japonica*	Ivory Coast	IRRI
231				Wells	*O. sativa*	*tropical japonica*	United States	CU
232				YRL-1	O. sativa	*tropical japonica*	Australia	IRRI

B. *O. rufipogon* accessions

S.No	IRGC ID	NSF ID	GSOR ID	Accession name	Species	Subpopulation	Country	Institute
233	80742	NSFTV407		407	*O. rufipogon*	W1	Myanmar	CU
234	105567	NSFTV490		490	*O. rufipogon*	W1	Indonesia	CU
235	105942	NSFTV512		512	*O. rufipogon*	W1	Thailand	CU
236	106412	NSFTV539		539	*O. rufipogon*	W1	Vietnam	CU
237	105349	NSFTV482		482	*O. rufipogon*	W2	India	CU
238	105375	NSFTV483		483	*O. rufipogon*	W2	Thailand	CU
239	100211	NSFTV666		666	*O. rufipogon*	W2	India	CU
240	106272	NSFTV575		575	*O. rufipogon*	W3	Papua New Guinea	CU
241	106273	NSFTV576		576	*O. rufipogon*	W3	Papua New Guinea	CU
242	106276	NSFTV579		579	*O. rufipogon*	W3	Papua New Guinea	CU
243	105491	NSFTV488		488	*O. rufipogon*	W4	Malaysia	CU
244	100897	NSFTV677		677	*O. nivara*	W4	India	CU
245	101967	NSFTV691		691	*O. nivara*	W4	India	CU
246	105599	NSFTV737		737	*O. nivara*	W4	Thailand	CU
247	106148	NSFTV757		757	*O. nivara*	AdmixW4/W1	Laos	CU
248	81909	NSFTV415		415	*O. spontanea*	W5	India	CU
249	93188	NSFTV444		444	*O. nivara*	W5	Nepal	CU
250	102178	NSFTV607		607	*O. nivara*	W5	India	CU
251	105400	NSFTV485		485	*O. rufipogon*	W6	China	CU
252	100907	NSFTV604		604	*O. rufipogon/O. nivara*	W6	Taiwan	CU
253	100593	NSFTV669		669	*O. nivara*	W6	Taiwan	CU
254		NSFTV763		763	*O. rufipogon*	W6	China	CU
255	W1944*	NSFTV766		766	*O. rufipogon*	W6	China	CU

C. Wild rice accession from different species

S.No	IRGC no. or Genebank ID*	NSF ID	GSOR ID	Accession name	Species	Subpopulation	Country	Institute
256	105220	NSFTV479		479	*O. officianalis*	outgroup	Indonesia	CU
257	105564	NSFTV489		489	*O. meridionalis*	outgroup	Indonesia	CU
258	W2121*			OR44	*O. meridionalis*	outgroup	Australia	CU
