# Supplementary material for: Large-scale deployment of a rice 6 K SNP array for genetics and breeding applications
Source: Rice (N Y). 2017 Aug 30;10:40. doi: 10.1186/s12284-017-0181-2 (PMC5577349; doi:10.1186/s12284-017-0181-2)
Supplement: Supplementary file 4 — Principal Components Analysis of diverse cultivated Asian rice accessions: PCA of 232 O. sativa accessions (PDF 353 kb) [file 12284_2017_181_MOESM4_ESM.pdf]

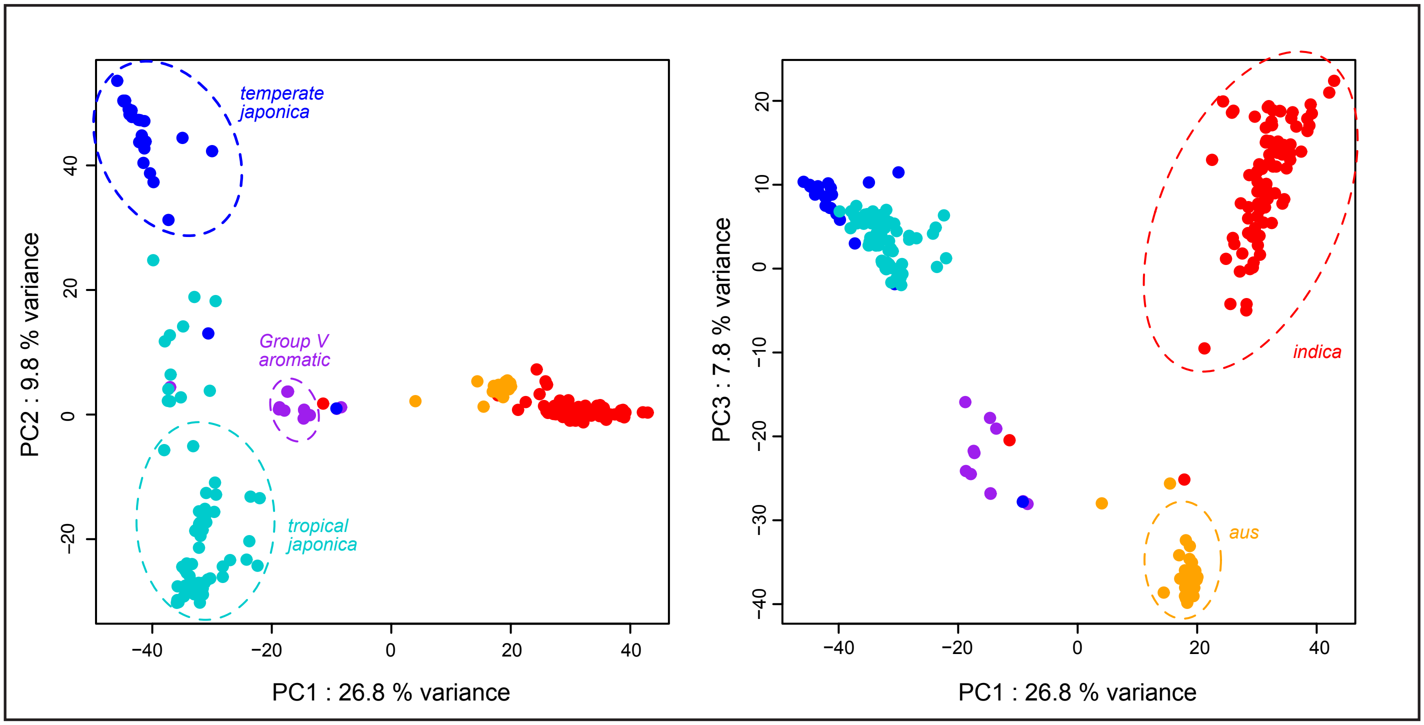

Figure S3. Principal Components Analysis of diverse cultivated Asian rice accessions. PCA of 232 *O. sativa* accessions. Colors indicate previously identified subpopulation membership (dark blue = *temperate japonica*, cyan = *tropical japonica*, purple = *aromatic*, red = *indica*, orange = *aus*). PC1 distinguishes the *Japonica* Varietal Group and the *Indica* Varietal Group, PC2 separates the *temperate japonica, tropical japonica* and *aromatic* populations; PC3 differentiates *indica* and *aus*.
