# Supplementary material for: Large-scale deployment of a rice 6 K SNP array for genetics and breeding applications
Source: Rice (N Y). 2017 Aug 30;10:40. doi: 10.1186/s12284-017-0181-2 (PMC5577349; doi:10.1186/s12284-017-0181-2)
Supplement: Supplementary file 5 — Use of C6AIR for background selection during TR22183-Pup1 development (PDF 476 kb) [file 12284_2017_181_MOESM5_ESM.pdf]

Figure S4. Use of C6AIR for background selection during TR22183-Pup1 NIL development

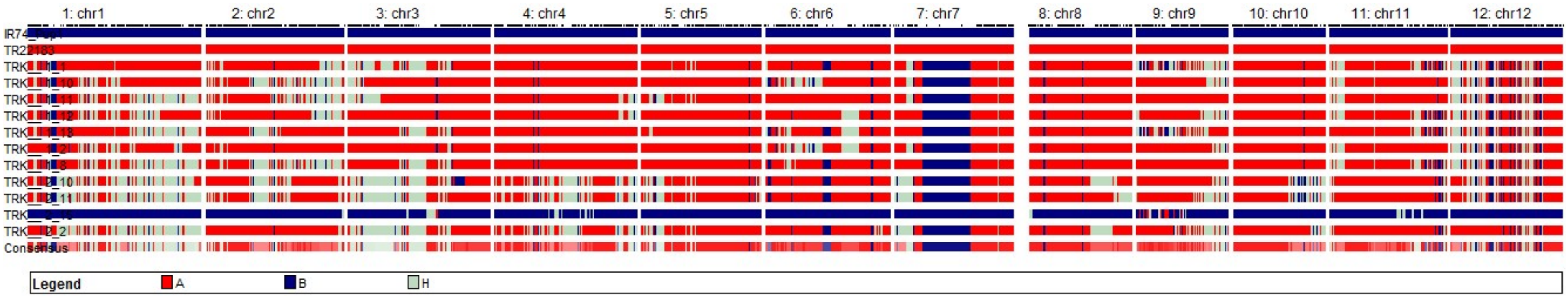

Graphical genotypes of selected BC2F1 of TR22183xIR74-Pup1 using 2055 polymorphic SNP markers. All selected plants were subjected to foreground genotyping using Pup1 major markers. Red color (A) indicates TR22183 segments, blue color (B) indicates donor introgression while light blue color (H) indicates heterozygosity.

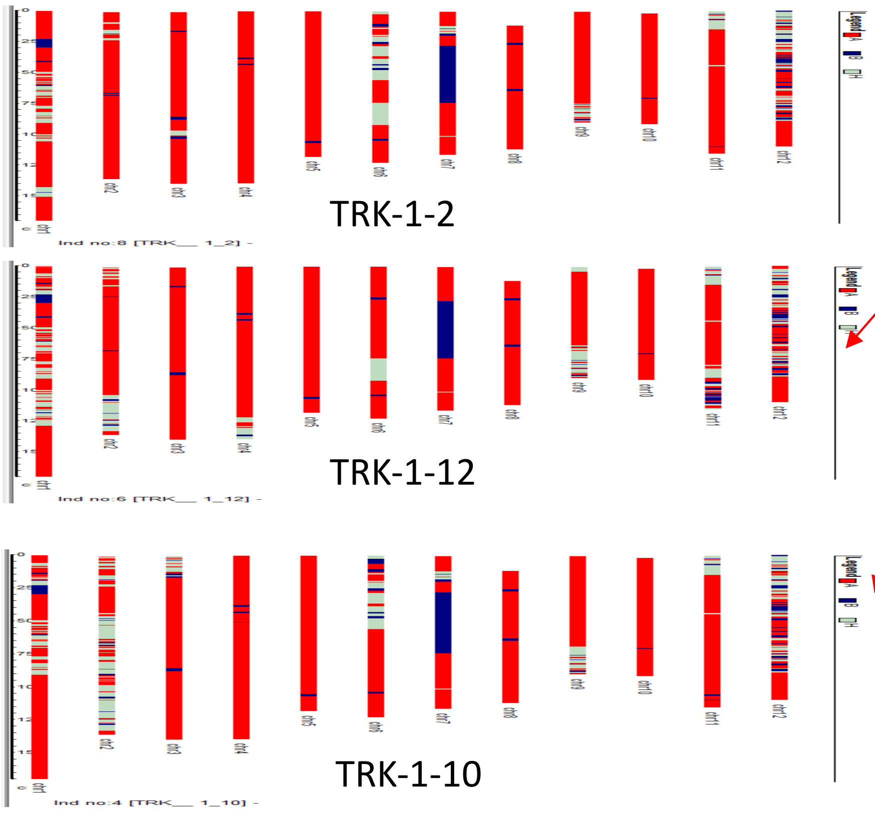

Graphical genotypes of the top 3 best BC2F1 plants in terms of percent recipient segment recovered.

Alias	A (%)	B (%)	H (%)	Total (cM)	Recombin	H-segments
TR22183	100	0	0	1438.7	0	0
TRK-1-2	81.9	7.1	11	1438.7	230	84
TRK-1-12	78.7	7.5	13.8	1438.7	284	107
TRK- 1-10	77.3	7.8	15	1438.7	303	118
TRK- 1-1	75.5	8.9	15.5	1438.7	344	132
TRK-1-11	75.2	7.3	17.5	1438.7	320	131
TRK- 1-13	75.1	8.8	16.1	1438.7	324	121
TRK-1-8	74	8.2	17.9	1438.7	323	126
TRK-2-2	67.5	7	25.6	1438.7	355	148
TRK-2-6	63.1	8.7	28.2	1438.7	430	182
TRK-2-7	61.6	9.2	29.2	1438.7	456	193
TRK-2-10	61.5	9.7	28.7	1438.7	435	181
TRK-2-11	61.1	9	29.9	1438.7	454	192
TRK-2-3	58.4	9.9	31.7	1438.7	434	181
TRK-2-8	54.7	9.5	35.9	1438.7	506	216
TRK-2-15	1	95.8	3.3	1438.7	85	32
IR74_Pup1	0	100	0	1438.7	0	0

Genotypic summary statistics of the selected BC2F1.
