# Supplementary material for: Large-scale deployment of a rice 6 K SNP array for genetics and breeding applications
Source: Rice (N Y). 2017 Aug 30;10:40. doi: 10.1186/s12284-017-0181-2 (PMC5577349; doi:10.1186/s12284-017-0181-2)
Supplement: Supplementary file 7 — Distribution of the frequency of “no call” (NC) alleles in (a) cultivated and (b) wild accessions using the Cornell 6 k Array Infinium Rice (PDF 1061 kb) [file 12284_2017_181_MOESM7_ESM.pdf]

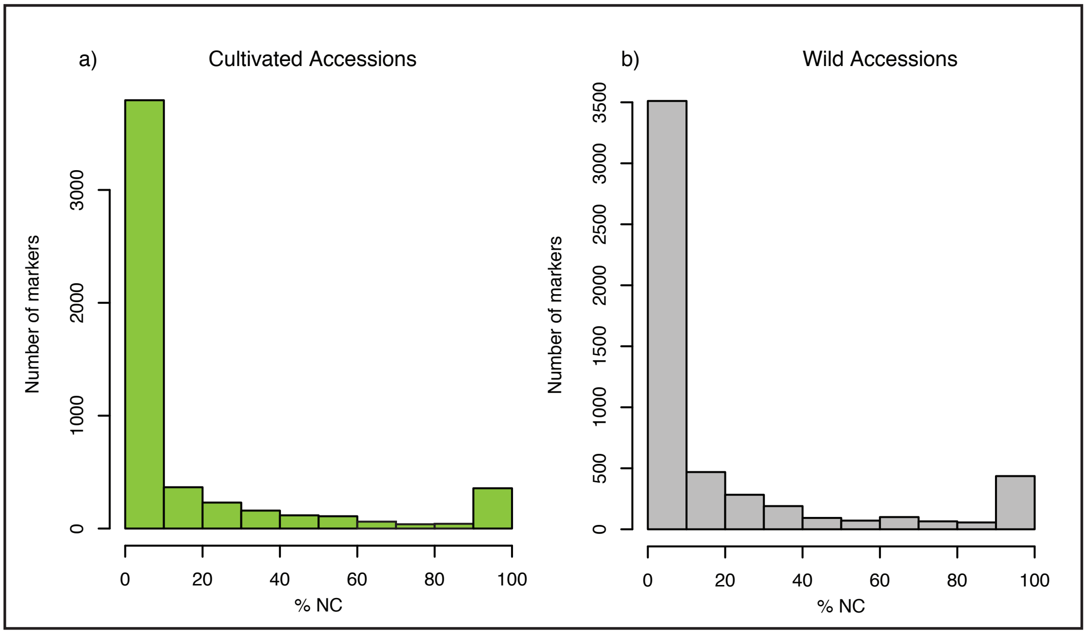

Figure S5. Distribution of the frequency of "no call" (NC) alleles in (a) cultivated and (b) wild accessions using the Cornell 6k Array Infinium Rice. There were 401 markers that have > 79% NC in cultivated and 472 markers in wild accessions. Both these groups shared 379 markers that gave > 79% no calls.
